# Influence of Gamma Irradiation on Different Phytochemical Constituents of Dried Rose Hip (*Rosa canina* L.) Fruits

**DOI:** 10.3390/molecules27061765

**Published:** 2022-03-08

**Authors:** Manol Ognyanov, Petko Denev, Desislava Teneva, Yordan Georgiev, Sabina Taneva, Iskra Totseva, Mariana Kamenova-Nacheva, Yana Nikolova, Svetlana Momchilova

**Affiliations:** 1 Laboratory of Biologically Active Substances-Plovdiv, Institute of Organic Chemistry with Centre of Phyto-chemistry, Bulgarian Academy of Sciences, 139 Ruski Blvd., 4000 Plovdiv, Bulgaria; petkodenev@yahoo.com (P.D.); desislava.teneva@orgchm.bas.bg (D.T.); yordan.georgiev@orgchm.bas.bg (Y.G.); 2 Laboratory of Lipid Chemistry, Institute of Organic Chemistry with Centre of Phytochemistry, Bulgarian Academy of Sciences, 9 Acad. Georgi Bonchev Str., 1113 Sofia, Bulgaria; sabina.taneva@orgchm.bas.bg (S.T.); iskra.totseva@orgchm.bas.bg (I.T.); svetlana.momchilova@orgchm.bas.bg (S.M.); 3 Laboratory of Organic Synthesis and Stereochemistry, Institute of Organic Chemistry with Centre of Phytochemistry, Bulgarian Academy of Sciences, 9 Acad. Georgi Bonchev Str., 1113 Sofia, Bulgaria; mariana.nacheva@orgchm.bas.bg (M.K.-N.); yana.nikolova@orgchm.bas.bg (Y.N.)

**Keywords:** gamma irradiation, rose hip, seed oil, polysaccharides, carbohydrates, lipids

## Abstract

Gamma irradiation is efficiently applied to many foods, but nevertheless there is a distinct lack of information about the changes of macro- and micronutrients (e.g., carbohydrates, lipids, organic acids, and phenolics) in dried rose hip (RH) fruits. Therefore, in this study, for the first time, the effect of gamma irradiation (10 and 25 kGy) on RH constituents is investigated. Different analytical techniques (GC-FID, HPLC-UV, HPSEC-RID, IR-FT, and SEM) are employed to examine this effect. The irradiation treatment (10 kGy) increased the glucose content by 30% and released cellobiose from RH fruits, thus revealing cellulose destruction. The extractability of total uronic acids increased from 51% (control) to 70.5% (25 kGy-irradiated), resulting in a higher pectin yield (10.8% < 12.8% < 13.4%) and molecular heterogeneity. Moreover, de-esterification was not a major effect of the irradiation-induced degradation of pectin. The sample exposure to the highest dose did not change the content of total carotenoids, *β*-carotene, and (un)saturated fatty acids, but it affected the tocopherols levels. Gamma rays had a negligible effect on the phenolic constituents and did not affect ORAC and HORAC antioxidant activity. In conclusion, it can be compromised that the exposition of dried RH is safe and can be successfully applied to decontaminate fruits without affecting their nutritional value and biological activity.

## 1. Introduction

The irradiation of foodstuffs with gamma rays was developed as a technology in the second half of the 20th century, and is nowadays among the most widely used approaches for microbial decontamination and the shelf-life extension of foods [1,2]. It is considered a safe, efficient, environmentally friendly, and energy-efficient method, and is particularly important in industrial food decontamination [3]. Preventing microbial spoilage by irradiating dried foods, such as fruits, herbs, spices, and nuts, at doses of 3–10 kGy is an important alternative to fumigation with microbicidal gases, however, doses above 20 kGy have also been used to reduce the microbial population in dry foods [4,5]. Along with the benefits of gamma ray treatment, adverse effects associated with changes in the chemical composition of irradiated foods can be expected. It is known, for example, that irradiation could have a negative effect on the structure of fatty acids in nuts or to affect the anthocyanin and polyphenol content of fresh fruits, such as pomegranates [6,7]. Due to conflicting results, scientists are still looking for fresh evidence to accept or reject the hypothesis about the positive or negative influence of irradiation on foods. Moreover, due to a great variety of foods with specific chemical compositions, each food must be examined individually.

Rose hip (RH) fruits (*Rosa canina* L.) are mainly used for producing jams, jellies, marmalades, tea, soups, food additives, and functional ingredients [8,9]. Although fresh RH fruits having the highest nutrient quality are particularly suitable for the production of various functional foods and food supplements, they are most often stored, transported, sold, and processed in a dried form. Some fruits’ characteristics appeared interesting enough to require a more detailed study of their changes after irradiation. Many studies, for example, provide empirical evidence to prove that RH is a rich source of vitamins, lipids, phenolic antioxidants, macro- and micro-elements, and polysaccharides (PSs) [8,9,10,11]. Moreover, because of their well-known beneficial health properties, the RH fruits are also used as the main ingredient in fruit teas [12]. In spite of this, to the best of our knowledge, studies on the effect of gamma irradiation on the changes in the chemical composition of dried RH have not been found.

Therefore, the present study aims to investigate the effects of gamma irradiation on the changes in the levels of different macro- and micronutrient constituents of dried RH fruits.

## 2. Results

### 2.1. Influence of Gamma Irradiation on Sugar and Organic Acid Composition

**Figure 1:**
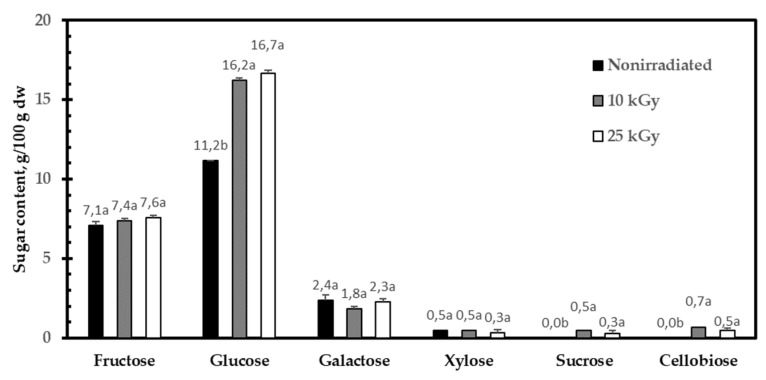
Sugar composition of non-irradiated, 10 kGy-, and 25 kGy-irradiated RH fruits. Different letters denote statistically different values, *p* < 0.05; dw—dry weight.

The changes in the content of free sugars in dried RH fruits caused by gamma irradiation are shown in Figure 1. It can be seen that glucose (53%) was the major sugar in non-irradiated fruits. Together with fructose (33%), it occupied more than 80% of the total free sugars, whereas galactose and xylose were very minor constituents. Surprisingly, the glucose content increased substantially by 30% (*p* < 0.05) with increasing the irradiation dosage, while the quantity of the other sugars seemed to be mainly unaffected by irradiation. It is interesting to note that this effect was particularly noticeable at a lower dose (10 kGy), and a further increase in the dose (25 kGy) did not have any considerable influence on the quantity of the glucose. Despite this, the irradiation influenced the pattern of sugar accumulation. One of the most interesting observations was that cellobiose was found in 10 kGy- and 25 kGy-irradiated fruits. The presence of cellobiose combined with an additionally liberated glucose amount presented a clear sign that a higher dose could inflict damage on the cellulose fibers that constituted the primary cell walls of plants. Additionally, the quantity of non-reducing sucrose was found to increase when RH fruits were irradiated (≥10 kGy). It was scarcely possible that a part of the starch/cellulose was converted into sucrose and glucose by affecting the carbohydrate-active enzymes.

Further, the effect of gamma irradiation on the organic acid profile was examined (Table 1). It should be mentioned that organic acids are a very important component of foods, which have a part to play in determining the taste of foods. As can be seen, citric acid, a member of the Krebs cycle, was predominantly present (2768 mg/100 g), because it occupied 55% of the total organic acids. Non-irradiated RH fruits also contained quinic acid (1344 mg/100 g) and ascorbic acid (624 mg/100 g) in smaller amounts, but, nevertheless, they accounted for almost 40% of the total organic acids content. From the data in Table 1, it is apparent that the exposition of fruits to gamma radiation has a minor or no effect on quinic, malic, and ascorbic acid content. It is evident that citric, *α*-ketoglutaric, and succinic acids increase in quantity. The increasing levels of these acids suggested a stimulation of respiration through the citric acid cycle, if we assume the presence of viable cells. However, the latter statement looked highly unlikely because the cell’s/tissue’s metabolism could be dramatically slowed down by moisture loss during freeze-drying (<10%). Thus, it is not wrong to assume that the cell walls and/or even (non)membrane-bound cell organelles (such as vacuole and mitochondrion) suffered extensive damage by irradiation, thus making small molecules more easily extractable. Further, the level of tartaric acid, a product of the oxidation of sugars, decreased slightly, but it was statistically significant at the highest level of radiation (25 kGy). An important point brought to mind is that organic acids and, above all, tartaric acid, have closely related metabolism to ascorbic acid [13]. It is possible that the level of tartaric acid was reduced in an attempt to compensate for the loss of ascorbic acid whose amount did not change significantly.

### 2.2. Influence of Gamma Irradiation on the Lipid Composition of Seed Oil

The results of the lipid composition of RH seed oil are presented in Table 2. Our experiment indicated that the yield of oil did not change considerably with irradiation (6.3–6.5%). Further, it can be seen that palmitic acid (16:0; 3.7%) and stearic acid (18:0; 2.2%) were the most abundant saturated fatty acids in the oil of non-irradiated RH fruits. Considering unsaturated fatty acid composition, seed oil was characterized by a high degree of unsaturation. Linoleic acid (18:2; 55%) represented the major polyunsaturated fatty acid constituent, followed by *α*-linolenic (18:3; 21%) and oleic acid (16:1; 16%). Despite this fact, it seemed that irradiation did not cause any significant changes in the fatty acid composition (Table 2), even though the sample was exposed to the highest dose (25 kGy). Considering the carotenoids, it was evident that neither the 10 kGy nor 25 kGy doses had any negative influence on *β*-carotene and, in general, on the total carotenoid content. It is worth mentioning that the total carotenoid content did not change significantly not only in seed oil, but also in fruit flesh (about 820 mg/kg) when the irradiation doses increased (Table 2). Our findings were in line with those of an earlier study that demonstrated that a 10 kGy irradiation of RH tea did not cause any change in the carotenoid content [14]. Further, one of the most interesting findings was that there was a noticeable difference in the amounts of *α*- and *γ*-tocopherol as a result of irradiation (*p* < 0.05) (Table 2). It is well known that tocopherols (vitamin E) are powerful antioxidants able to scavenge free radicals. It may be that the tocopherols were reduced because of their active participation in the neutralization of free radicals.

**Figure 2:**
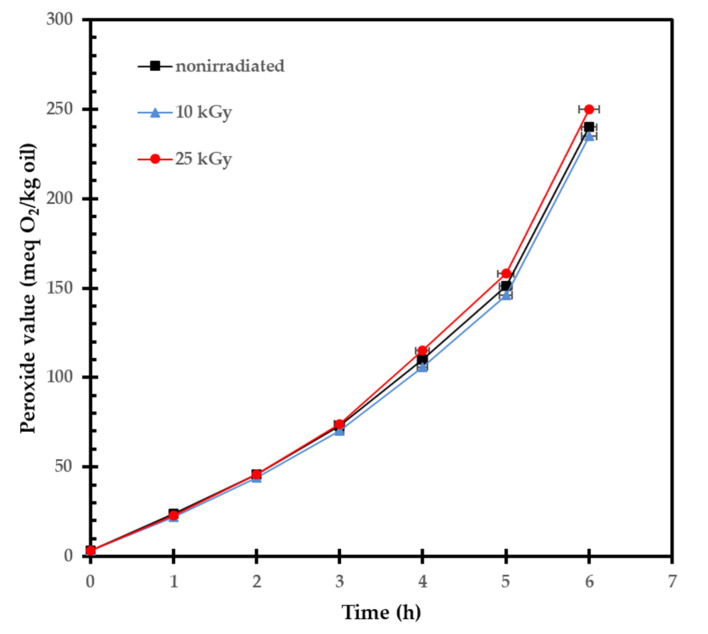
Induction periods of RH seed oils extracted from non-irradiated, 10 kGy-, and 25 kGy-irradiated RH fruits.

In addition, important parameters, such as the acid value, amounts of conjugated dienes, and trienes, were determined. These parameters are reliable indicators of the degree of lipid oxidation. The results obtained are also summarized in Table 2. Our findings indicated that the conjugated trienes, and especially dienes, were slightly influenced by a higher dose (25 kGy). The results obtained are broadly in line with the expectations, because unsaturated fatty acids comprised 93% of the total fatty acid content. In general, a higher amount of unsaturated fatty acids suggested a higher instability of oil due to oxidation. Therefore, the determination of the induction period of auto-oxidation of RH oil has a more practical significance when the degree of oxidation stability should be evaluated (Figure 2). It represents the time interval during which the antioxidants present in the sample still effectively inhibit the auto-oxidation processes in the oil, and the rate of formation and accumulation of hydroperoxides still does not increase exponentially. As illustrated in Figure 2, it seems that gamma irradiation (<25 kGy) does not significantly change the oxidative stability of RH seed oil because of the presence of antioxidants.

### 2.3. Influence of Gamma Irradiation on the Phenolic Constituents and Antioxidant Activity

The content of the total phenolics and in vitro antioxidant activity of non-irradiated, 10 kGy-, and 25 kGy-irradiated RH fruits was investigated. Together with the quantitative data for the phenolic components, they are included in Table 3.

In general, gamma irradiation had a negligible effect on the content of the phenolic compounds and their profile. As it is evident from the results, the total polyphenol content shows a trend towards increase, however without reaching significant differences (*p* < 0.05). The only significant difference was observed in the quercetin content, which increased from 13.9 mg/100 g to 18.9 mg/100 g, and 18.3 mg/100 g for fruits irradiated with 10 kGy and 25 kGy, respectively (*p* < 0.05). On the other hand, the detected amounts of quercetin-3-*O*-glucoside and rutin decreased dose-dependently after irradiation (*p* > 0.05), which explained the increase in quercetin content and could be attributed to the cleavage of glycosidic bonds from the high-energy electromagnetic radiation [15]. Gamma irradiation showed no effect on the amounts of chlorogenic acid, catechin, and epicatechin in RH fruits. Even though quercetin is a stronger radical scavenger than its glycosides, the antioxidant activity measured by the ORAC and HORAC assays did not change as a result of the treatment.

### 2.4. Influence of Gamma Irradiation on Polysaccharide Constituents

We were very interested in knowing more about the potential changes in different PSs because they are the most important plant cell wall constituents. The results of different analyses are summarized in Table 4. From the table, it is evident that the yield of alcohol-insoluble solids (AISs) decreases with increasing the radiation dose (0 > 10 > 25 kGy). Accordingly, this means having a higher proportion of alcohol-soluble small molecules as a result of irradiation (0 < 10 < 25 kGy). These results provide us with the next clear evidence to support our assertion about the damage of plant cell wall constituents during irradiation. These cell wall changes should facilitate the extraction of initially present or additionally formed (as a result of irradiation) low molecular weight alcohol-soluble compounds (such as sugars and oligomers). It is interesting to mention that the yield of PS also increased significantly when RH fruits were exposed to higher doses of radiation: non-irradiated—10.8%, 10 kGy—12.8%, and 25 kGy—13.4%. These results suggest that some cell wall constituents are adversely affected by irradiation, thus directly contributing to the additional release of PS populations. The latter results are very closely related to the finding of a considerable percentage of uronic acids extracted from the fruits as a result of irradiation (Table 4; control—51% and 25 kGy—71%). It is well known that uronic acids are major constituents of pectic PSs and, together with hemicelluloses, mainly comprise the primary cell walls of plants. Table 4 shows that the 10 kGy- and 25 kGy-irradiation does not exert any changes in the amount of the total uronic acid in the initial RH fruits (9.5–10.5%). At first sight, it can be interpreted to mean a ‘lack of changes in pectin’. The PSs obtained were characterized as typical high-methyl-ester pectins having a uronic acid content in the range between 49 and 53%. Surprisingly, it seemed that the degree of methyl-esterification was not affected by radiation (Table 4; 69–70 mol%), strongly suggesting that de-esterification is not a major effect of the irradiation-induced degradation of pectic PS. However, the degree of acetylation tended to be increased with increasing the irradiation dose. It may be that PS fractions carrying a higher number of acetyl groups were additionally released as a result of irradiation. Further, it could be observed that irradiation exerted a profound influence on cellulose that led to a reduction in its amount (Table 4). This was a key finding, which directly confirmed our above-mentioned statement about cellulose destruction. It could be argued that a minor part of the cellulose skeleton was broken down into cellobiose, low-molecular-weight cello-dextrins, or other oligomers (alcohol soluble), thus causing a reduction in the cellulose level and a rise in the yield of alcohol-soluble solids. Taken together, the above results suggest that a part of the pectin was trapped in the (hemi)cellulose network, and by degrading a part of the cellulose segments, additional pectic PSs were released leading to a rise in the yield of PSs. An interaction between pectin and cellulose was demonstrated in a previous study [16]. In fact, it should not rule out the possibility that the gamma radiation caused a direct degradative action on the pectin, in addition to that of cellulose.

#### 2.4.1. Influence of Gamma Irradiation on Monosaccharide Composition

Table 5 shows the results of the monosaccharide composition analysis of non- and 25 kGy-irradiated PSs fraction. It can be seen that commonly reported pectic monosaccharides constitute the PS fractions. The PS isolated from non-irradiated fruits was composed mostly of galacturonic acid, glucose, and rhamnose. Other monosaccharides, such as galactose and arabinose, were present in lower amounts. Irradiation did not have a considerable influence on different sugar constituents. It appears that gamma-irradiation contributes only to the reduction in the glucose content, whereas that of fucose is raised, possibly due to changes in the extractability of PSs and plant cell walls.

#### 2.4.2. Influence of Gamma Irradiation on Molecular Weight Distribution

**Figure 3:**
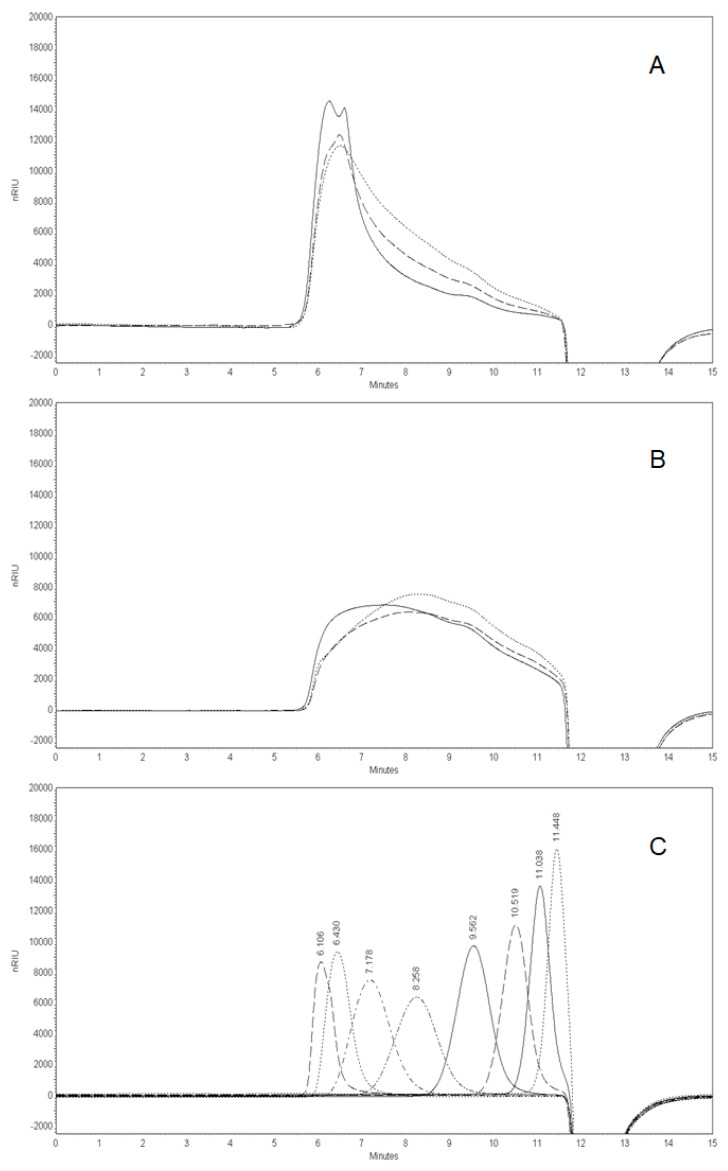
High-performance size-exclusion chromatography (HPSEC) elution pattern of PSs isolated from non-irradiated, 10 kGy-, and 25 kGy-irradiated RH fruits as: (**A**) initial PSs; (**B**) *endo*-PG-degraded PSs (solid—non-irradiated; dash—10 kGy; dot—25 kGy); and (**C**) Pullulan standards (0.59–78.8 × 10^4^ g/mol) were used to estimate the molecular weights.

The molecular weight distribution pattern of the isolated PSs fraction is shown in Figure 3A. It can be seen that the PS fraction, isolated from non-irradiated fruits, consists mainly of two high molecular weight populations covering the range between 78.8 × 10^4^ and 21.2 × 10^4^ g/mol (Figure 3A solid line). Moreover, the main peak is accompanied by a pronounced tailing suggesting that heterogeneity exists within the sample. It ranges between 21.2 × 10^4^ and 4.73 × 10^4^ g/mol. What is immediately striking, from a close inspection of Figure 3A, is that irradiation causes the PS molecular weight elution pattern to change visibly. A 10 kGy-irradiation leads to a decrease in the main peak intensity (78.8 × 10^4^ g/mol), suggesting that part of the high molecular weight populations is converted into lower molecular weight compounds (Figure 3A dash line). As a consequence, the increase in the area representing medium and low molecular weight fractions is found (RT > 7.1 min). Interestingly, this negative effect on the PSs became stronger when the sample was exposed to the highest dose (25 kGy), and thus resulted in an even greater increase in the area representing lower molecular weights. More specifically, it is easy to be observed that, if we integrate the chromatograms (Figure 3A), the high molecular weight populations (RT 5.5–7.0 min) occupied a smaller percentage of the total (100%) peak area, hence a smaller percentage of PSs, when the irradiation dose increased: 0–60%, 10 kGy—48%, 25 kGy—40%. Accordingly, the percentage of lower molecular weight populations (RT 7.0–11.6 min) that comprised the PSs jumped: 0–40%, 10 kGy—52%, 25 kGy—60%.

To receive more information about the structural features of PSs, an *endo*-polygalacturonase (*endo*-PG) hydrolysis of the PS fraction was performed. It is a well-known fact that *endo*-PG can cleave *α*-1,4 glycosidic bonds between two non-esterified galacturonic acid units in the homogalacturonan (HG) region of pectin [17]. Figure 3B illustrates the elution pattern of *endo*-PG-treated samples. It can be observed that the *endo*-PG carried out the conversion of the part of the high molecular weight material into a broad range of lower molecular weight compounds. As a result, the change of the elution profile was observed by comparison with the non-enzyme-treated PS (Figure 3A solid line). This presented a reasonable reason to suppose that the HG region of pectin was adversely affected by gamma irradiation. A surprising finding of this study was that the PSs, isolated from 10 kGy- and 25 kGy-irradiated fruits, were degraded by *endo*-PG more efficiently than those isolated from the non-irradiated control. This led to an additional increase in the peak area and shift in the retention time reflecting the accumulation of a large number of lower molecular weight compounds (Figure 3B dash and dot line).

These results could be expressed in numerical data, if we integrate the chromatograms (Figure 3B). It was precisely calculated that the percentage of the total peak area ascribed to high molecular weight compounds (RT 5.5–7.0 min) decreased by 7%: 0–23%, 10 kGy—18%, 25 kGy—16%. Accordingly, the area percentage of lower molecular weight populations (RT 7.0–11.6 min) increased by 7%: 0–77%, 10 kGy—82%, 25 kGy—84%.

This finding can be interpreted in different ways. On the one hand, it is known that *endo*-PG requires a sequence of 2–3 unesterified galacturonic acid units to cleave them [17]. However, keeping in mind that the irradiation did not cause any appreciable effect on the degree of methyl-esterification (Table 5), we can suppose a difference between the esterification pattern of the PSs. On the other hand, it may be that lower molecular weight HG segments were a better substrate for *endo*-PG action.

#### 2.4.3. Fourier Transformed Infrared (FT-IR) Spectroscopy

**Figure 4:**
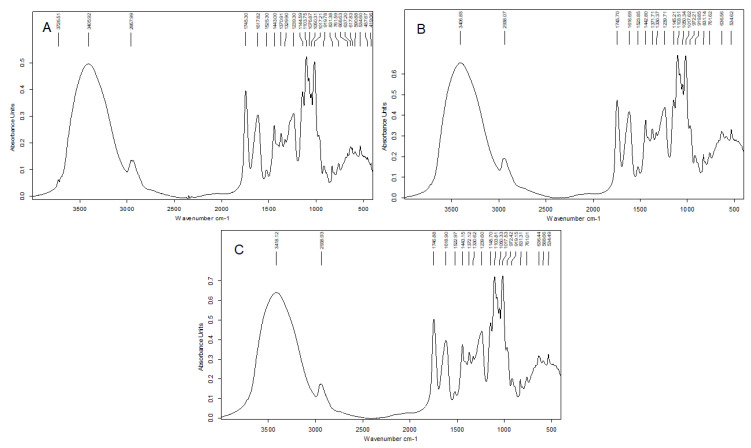
FT-IR spectra of the PSs isolated from non-irradiated (**A**), 10 kGy- (**B**), and 25 kGy-irradiated (**C**) RH fruits.

The FT-IR spectra of the studied PSs are presented in Figure 4. FT-IR can identify the presence of specific functional groups prevailing in the sample. The typical bands for pectic PSs were found [18]. The broadened band that occurs in the region ranging from 3730 to 3100 cm^−1^ was assigned to the ν(OH) stretching vibrations of free hydroxyl groups involved in intra- and intermolecular H-bonding. The band at 2940–2960 cm^−1^ was attributed to the ν(CH) stretching of CH_2_ groups. The presence of a well-separated sharp signal that appears at 1745–1735 cm^−1^ was typical for the C=O stretching vibration of methyl-esterified carbonyl groups of pectic PSs (ν_(C=O)_COOH). The bands at 1630 and 1438 cm^−1^, together with that at 1440 cm^−1^, were due to the asymmetric ν_as_(O=CO–) and symmetric ν_s_(O=CO–) stretching vibrations of the non- and esterified carboxyl groups, respectively. The signals at 1523–1525 cm^−1^ could originate from the aromatic ring vibrations of residual co-extracted phenolics in PSs because their protein content was low (Table 4). The presence of a band at 1238 cm^−1^ corresponded to –C–O stretching of the acetyl residue, which was consistent with a higher value of the degree of acetylation (Table 4). In addition, bands at 1145 cm^−1^, 1101 cm^−1^, and 1018 cm^−1^ could be interpreted with the glycosidic bond vibrations involving ν(C–OC), –ν(CC)(CO), and –δ(O=C–H) bending in the pyranose ring. The C1-OH vibrations of the monosaccharide ring at 831 cm^−1^ showed the presence of *α*-anomeric configuration, which is typical for galacturonic acid in pectins.

It was apparent that the characteristic infrared bands neither disappeared from the spectra nor appeared new ones. At first sight, according to these spectra, there was no reason to suppose that gamma irradiation caused a negative effect on the chemical composition of pectic PS. It is possible for the changes to have been minor, as they could not be seen in the spectra. However, an increase in the band intensities suggested some changes in the amorphous state of PSs, as demonstrated for starch.

### 2.5. Scanning Electron Microscopy (SEM) of the Fruit Material

**Figure 5:**
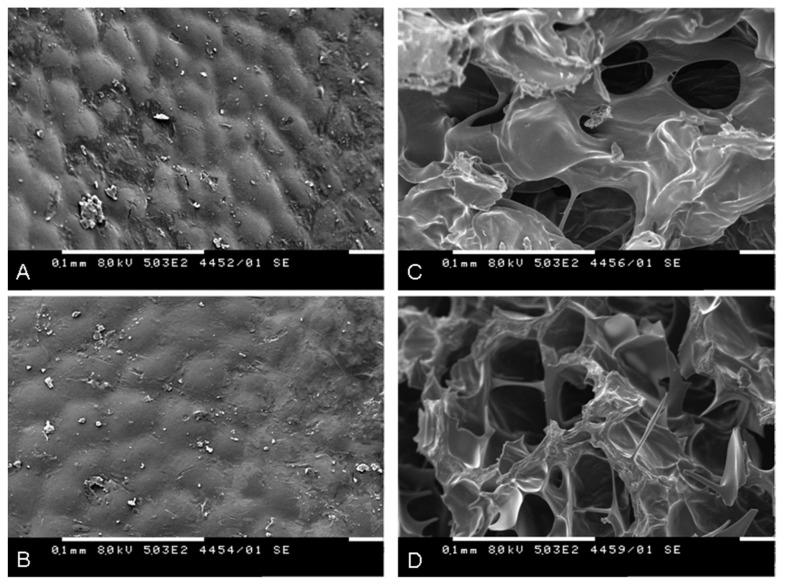
SEM images of the surface of non-irradiated (**A**) and 25 kGy-irradiated (**B**) RH fruit, and the internal part of non-irradiated (**C**) and 25 kGy-irradiated (**D**) RH fruit (8 kV, 0.1 mm, 500×).

If the gamma irradiation of the fruits caused severe damage to the components of the plant cell walls and tissues, they would be reflected in a change in the surface morphology of the plant material. Therefore, we examined the surface and internal parts of non-irradiated and 25 kGy-irradiated RH fruit material. Figure 5 shows the generated images of the internal and external parts of the fruits. No characteristic changes in the morphology of the surface (exocarp) of the irradiated sample were observed, despite the fact that a very large part of the surface was carefully inspected (Figure 5A,B). However, the examination of the internal part (mesocarp) of the 25 kGy-irradiated fruits revealed that the inner walls became less thick, and the cavities were enlarged (Figure 5C,D). Nevertheless, a more precise histochemical test must be performed to help confirm the latter statement.

## 3. Discussion

In general, gamma irradiation is a low-cost technology for the sterilization and decontamination of foods. However, together with the positive results, irradiation brings about a negative impact on the nutritional and functional properties of the foods. This effect is strongly determined by the received irradiation dose [3]. The quality of food depends upon the chemical composition (for instance, water, lipids, proteins, and PSs), and thus the changes in the level of constituents have an immediate impact on them. Therefore, in the current study, RH was investigated in detail to determine whether the gamma-irradiation processing contributed to the changes in the phytochemical constituents of the fruits.

### 3.1. Influence of Gamma Irradiation on Carbohydrate Constituents

Our research findings indicated that the carbohydrate constituents of RH fruits were among the most seriously affected by irradiation. Irradiation contributed to an increase in the reducing sugars content (mainly glucose), which can be ascribed to the structural breakdown of cellulose and, to a lesser extent, starch. A similar effect of gamma radiation on reducing the sugar content was observed in a previous study on the irradiation of dates [19]. According to these authors, irradiation (0.9–1.8 kGy) contributed to a high level of glucose, but it could not bring about a significant change in the fructose content. Moreover, scientists highlighted the fact that the decrease in starch content is paralleled by an increase in the glucose amount [19]. Our results on cellulose and glucose content follow a similar pattern. It is useful to recall that cellulose is a glucose-containing polymer organized into stable microfibrils with crystalline and amorphous regions. Cellulose meshes into a cell wall polymer matrix together with pectin, lignin, and hemicellulose. More importantly, cellulose fibers play a crucial role, mainly in maintaining the strong mechanical resistance of plants [20]. Therefore, it can be safely predicted that irradiation (≥10 kGy) leads to a change in the mechanical properties of dried RH fruits. It is interesting to speculate whether the strength of this effect can be increased after the pectic backbone also suffers serious damage as a result of irradiation. Pectin is an amorphous heteropolysaccharide found in the middle lamella, which is composed of the HG, rhamnogalacturonan-I, and rhamnogalacturonan-II regions. HG is the main pectic structural fragment that only consists of an unbranched chain of *α*-1,4-linked galacturonic acid units. These residues can be methyl-esterified and/or acetyl-esterified. Our results strongly suggest that RH pectin also mostly consists of HG (Table 5; ≈50 mol%). For that reason, together with a decrease in the molecular weight before and after *endo*-PG action (Figure 3A,B), we assumed HG to be mainly affected. The depolymerization of HG is a clear sign pointed to contribute to an undesired defect of RH fruits. Further, monosaccharide composition analysis revealed that gamma irradiation contributed only to the reduction in the glucose content, while that of fucose was raised. Glucose, in this regard, is not a typical constituent of pectin. However, this observation could be explained by the decomposition of very closely cross-linked pectin and cellulose complexes. Apart from that, it can be speculated that a higher amount of fucose in 25 kGy-irradiated PS could be due to an increased extractability of fucose-containing rhamnogalacturonan-II segments of pectin.

The degradation of cellulose and pectin by gamma irradiation was investigated in previous studies [21,22,23]. None of these studies, however, investigated the changes in polymer nature directly in the plant material. In contrast, observations of the effects of radiation were performed on purified dry substances and/or sugar-containing solutions (for pectin). As a sign of degradation, authors mainly used the changes in the viscosities of the solution of the irradiated samples. Concerning cellulose, the authors’ findings suggested that crystallinity and the amorphous region were changed due to additional oxidation seen by a decrease in the degree of polymerization and an increase in the absorbance of the carbonyl groups band at 1730–1750 cm^−1^ [21,23]. It should be noted that the alteration in cellulose was observed at dosages (100–2000 kGy) far exceeding ours (10 and 25 kGy). As regards the pectin, an increase in the degree of heterogeneity and a decrease in specific viscosity of 1% citrus pectin solution occurred on irradiation at a high dose level (20.56 kGy) [24]. Other scientists demonstrated by viscosity measurements and jelly tests that, unlike dried pectin, pectin in solution degraded more rapidly by irradiation (10–40 kGy). Interestingly, the jellies were not affected [25]. Our findings concerning the changes in the molecular weights were in accordance with another study that dealt with apple pectin exposed to 0.5–10 kGy of gamma radiation. On the contrary, the degree of esterification was a sharp contrast to our result [26]. However, our result is in agreement with a previous study on the examination of pectin solution exposed to 0.1–1.0 kGy of radiation, where any changes in methanol content were not confirmed [27]. It can be concluded that the differences that can be observed with the other results lie in the way the plant material is prepared (fresh or dried), the amount of carbohydrate constituents, and last but not least the irradiation doses applied.

### 3.2. Influence of Gamma Irradiation on Organic Acids

Concerning organic acids, we found evidence to suggest that the exposition of RH fruits to gamma radiation had a negligible effect on the content of quinic, malic, and ascorbic acids. However, most previous studies showed that ascorbic acid was highly susceptible to irradiation. It has been noted that an oxidized dehydroascorbic acid was formed as a direct result of irradiation. For example, the exposition of dried lycium fruit to gamma irradiation (2–14 kGy) had a devastating effect on ascorbic acid at higher doses (14 kGy)—close to the used in the current study [28]. An appreciable loss of ascorbic acid was also found in spice and herb extracts of black pepper, cinnamon (32%), nutmeg (48%), oregano, and sage after being exposed to irradiation (10 kGy) [29]. However, another study indicated that vitamin C content had not been dramatically affected when using a lower dose of radiation (0.15–1 kGy) [30]. The thing to remember is that ascorbic acid is a major water-soluble antioxidant molecule in RH fruits, and therefore the lack of change in its amount was an important finding for the preservation of the quality of treated fruits. Furthermore, it has little information about the changes in the citric and malic acid contents [31]. These authors linked the changes in the citric acid content of fresh potatoes exposed to irradiation (150 Gy) to the storage temperatures and time. A marked increase in citric acid content, for example, was observed at the sixth month of storage, whereas malic acid did not significantly change [31]. It should be noted that great care is needed when interpreting these results, because a large variety of experimental patterns occur.

### 3.3. Influence of Gamma Irradiation on Lipid Constituents

Our results reveal that irradiation at doses 10 and 25 kGy have no significant effect on the RH seed fat content, fatty acid composition, and carotenoids amount. Although a slight increase in the acid value, conjugated dienes, and trienes, together with a decrease in the tocopherols were detected, the oxidative stability of oil presented by the induction period of autoxidation was not practically changed. The scientific literature on the topic was not full of examples of irradiated RH fruits and seed oil in particular, and thus to be compared with our results. To date, to our knowledge, only one study, which has not specifically dealt with RH fruits, demonstrated that the carotenoid content of RH tea was not influenced by a dose of up to 10 kGy, probably due to the very low moisture content [14]. Our results generally support this view. It all points to the conclusion that not only plant sources, but also irradiation and post-irradiation storage conditions (such as temperature, light, and oxygen) were all important factors in cases of different lipid compositions.

### 3.4. Influence of Gamma Irradiation on Phenolic Constituents and In Vitro Antioxidant Activity

Our study provided evidence to prove that gamma irradiation did not have a dramatic effect on the content of the phenolic components of dried RH fruit. However, it is a known fact that gamma irradiation can affect the number of antioxidant components in plant foods. It could be attributed either to the increased ability of a plant to produce radioprotective antioxidants, or the increased extractability of phenolic antioxidants from the degraded plant matter [32]. The literature contains some observations on the effect of gamma irradiation on the antioxidant activity and polyphenol content of food matrices, but researchers found conflicting results. For example, the total polyphenol content and antioxidant activity of pomegranate peel powder and dried apricots increased as a result of irradiation, whereas no changes were observed in navel oranges [33,34,35]. The effect of gamma rays on foodstuffs depends on many factors, such as the radiation dose, moisture content, and the type and amount of endogenous polyphenols [32]. The enhanced antioxidant activity of irradiated plant material was mainly attributed to the increased enzyme activities or to the increased extractability from degraded plant tissues. On the other hand, gamma irradiation could reduce the antioxidant activity, which can be ascribed to the irradiation-derived degradation components or the formation of free radicals [32]. In our study, despite the degradation of dried RH matter, no significant changes in both the polyphenol content and the antioxidant activity of the treated samples were observed. This can be attributed to the lower moisture content of RH, which drastically limited enzymes’ activity, to the absence of the oxidative changes in irradiated fruits, and/or the impossibility of polyphenols taking part in the process of inactivating free radicals. This effect was in agreement with the findings of other authors, who observed that the total polyphenols in linden flowers did not alter during irradiation (<10 kGy) [14].

## 4. Materials and Methods

### 4.1. Fruit Material

The RH fruit material (an orange-red color; reached maturity; 47% dry matter) was obtained from a local producer (Smolyan, the Rhodope Mountains, Bulgaria). The material was enclosed in polyethylene bags and stored frozen (−18 °C) before further freeze-drying (<10% moisture content).

### 4.2. Gamma Irradiation

Before irradiation, the fruit flesh was separated from the seeds mechanically. The samples were irradiated separately at a radionuclide ^60^Co source with 8200 Ci activity. The gamma rays facility has a mobile irradiation chamber with 4.0 L volume and dimensions: 13.5 cm diameter and 22 cm height. During the irradiation, the chamber rotates on its vertical axis. For the study of the absorbed dose, distribution Alanine dosimeters (Kodak BioMax) were used, measured by ESR spectrometer E-scan Bruker, and calibrated in units of absorbed dose in water. At each point, three dosimeters were placed. The chosen absorbed doses were 10 kGy and 25 kGy. The fruit flesh and seeds (control and irradiated samples) were kept in polyethylene bags at room temperature. The irradiation of samples was performed in the National Center of Radiobiology and Radiation Protection at the Ministry of Health (Sofia, Bulgaria). Irradiated fruits and seeds, together with non-irradiated (control) samples were subjected to analyses immediately after irradiation.

### 4.3. Chemical Characterization of Non-irradiated and Irradiated Fruits

#### 4.3.1. Preparation of Alcohol-Insoluble Solids

RH fruits (non-, 10 kGy-, and 25 kGy-irradiated) without seeds were used for the preparation of AIS. For that purpose, the initially ground samples were incubated at 50 °C for 1 h with 70% (*v*/*v*) aqueous ethanol and then the solids were separated by centrifugation (18.187× *g*, 10 min, 5 °C). The same procedure was repeated 3 times. Finally, the residue was washed 2 times with acetone at room temperature and vacuum-dried until there was no change in the mass.

#### 4.3.2. Crude Protein Content

The crude protein content was evaluated by the micro-Kjeldahl method. The determination of nitrogen expressed as the ammonia content of the digested sample was performed by the acetylacetone–formaldehyde colorimetric method using ammonium sulfate as a standard [36]. The results were calculated using 6.25 as a conversion factor.

#### 4.3.3. Total Uronic Acid and Cellulose Content

The uronic acid content of the plant material was estimated as described [37]. As a sample for the analysis served AIS-prepared as described above (Section 4.3.1). In brief, the sample was suspended in 72% (*w*/*w*) H_2_SO_4_ (1 h, 30 °C), and after dilution with water to 1 M H_2_SO_4_, hydrolysis was achieved through further incubation for 3 h at 100 °C. An aliquot of hydrolysate was taken for an automated 3-phenylphenol colorimetric analysis using a continuous flow analyzer Skalar San^++^ system (Skalar Analytical BV, Breda, the Netherlands). The analysis was conducted according to the instructions of the manufacturer. Absorption was measured at 530 nm and galacturonic acid (12.5–100.0 μg/mL) was used for a calibration curve construction.

The quantitative estimation of cellulose in control and irradiated plant material was performed according to the gravimetric method of Kürschner and Hanak with a modification [38]. Briefly, a sample (0.5 g) was gently boiled (30 min) with 25 mL of acetic acid-HNO_3_ reagent (acetic acid: H_2_O:HNO_3_ 8:2:1 *v*/*v*/*v*) in a round-bottom flask fitted with a reflux condenser. After cooling the insoluble residue was filtered through a sintered glass filter (G3) under vacuum, washed with deionized water to neutral pH, then with ethanol (96% *v*/*v*), and finally with an excess of petroleum ether. The obtained residue was dried in a laboratory oven at 50 °C to a constant weight. The resulting cellulose was corrected for its ash content.

#### 4.3.4. Total Lipid Content

The total lipid content of the seeds was determined gravimetrically after an exhaustive extraction with *n*-hexane in a Soxhlet extractor (8 h) [39].

#### 4.3.5. Total Carotenoids, β-Carotene, and Tocopherol Content

For the estimation of the total carotenoid content of the fruit flesh, 1 mL methanol, 2 mL chloroform, and 1 mL of 50 mM Tris buffer (pH 7.5, containing 1 M NaCl) were added to 20 mg of the ground sample. The mixture was centrifuged (6000 rpm, 10 min), and after that, the chloroform-containing bottom phase was removed. The upper phase was re-extracted with 2 mL chloroform, and after centrifugation, two chloroform phases were combined, dried under a stream of nitrogen, re-dissolved in a mixture of cyclohexane and dichloromethane (9:1 *v*/*v*), and the absorbance of the solution was then measured at 445 nm (Cecil Series 8000 UV/VIS spectrophotometer, Cecil Instruments Ltd., Cambridge, UK). A calibration curve constructed with *β*-carotene was used for the quantification [40].

The quantities of *β*-carotene and tocopherols were performed by a high-performance liquid chromatography (HPLC) analysis of a hexane solution of oil. The analyses were conducted by Agilent 1100 apparatus equipped with a DAD detector operating at 292, 298, and 450 nm for the monitoring of *α*-, *γ*- tocopherols, and *β*-carotene, respectively, on a Nucleosil 100-5 (250 mm × 4.6 mm, 5 µm) column connected with a pre-column EC 4/3 Nucleosil 100-5 (Macherey-Nagel). The elution of tocopherols was performed with a mobile phase of hexane and tetrahydrofuran (96:4 *v*/*v*), at a flow rate of 1 mL/min [41], whereas *β*-carotene was eluted with a mixture of *n*-hexane and 2-propanol (98:2 *v*/*v*) at 1.0 mL/min. The identification of different analytes was performed by the comparison of retention times with those of individual standards, while the concentration was calculated using calibration curves.

#### 4.3.6. Conjugated Dienes and Trienes Contents

The conjugated dienes and trienes contents of RH seed oils were estimated by measuring the absorbance at 232 nm and 268 nm, respectively, of 1% solution of oil in *iso*-octane (Cecil Series 8000 UV/VIS spectrophotometer) with reference of pure solvent [42].

#### 4.3.7. Acid Value, Peroxide Value, and Induction Period

The acid value of RH seed oils was determined titrimetrically with ethanolic KOH [43]. The peroxide value, expressed as meq O_2_/kg oil, was determined according to the modified iodometric method of Yanishlieva et al. [44]. The induction period was evaluated as follows: oxidation of oil (2 g) was carried out at 100 °C by blowing a stream of air (50 mL/min). The peroxide value was estimated periodically at different intervals during oxidation and the increase in the value was monitored graphically. The induction period was determined by the method of Le Tutour and Guedon [45].

#### 4.3.8. Fatty Acid Composition

The fatty acid composition was determined by gas chromatography (GC) with a flame ionization detector after transesterification of oil to methyl esters with 1% H_2_SO_4_ in methanol [46]. Prior to the GC analysis, fatty acid methyl esters (FAMEs) were purified by thin-layer chromatography on a silica gel plate with a mobile phase composed of hexane-acetone (100:6 *v*/*v*). GC was conducted on a Shimadzu 17A apparatus coupled to a flame ionization detector using a Simplicity-wax (30 m × 0.32 mm × 0.25 μm, Supelco) column. The separation took place at the following temperature regime: from 170 °C, at 2 °C/min to 260 °C (5 min). The injector and detector temperatures were kept at 260 °C and 280 °C, respectively. Helium was used as a carrier gas at a flow rate of 0.5 mL/min and a split ratio of 1:50. The peak identification was according to retention times compared to that of a standard mixture of FAMEs.

#### 4.3.9. HPLC Determination of Free Sugars

About 1 g of the ground sample was extracted with a 3% solution of *meta*-phosphoric acid in water (30 mL) for 1 h at 30 °C shaking on a thermostatic water bath (NÜVE, Turkey). The residue and extract were separated through centrifugation (6000×*g*, 20 min) and an additional filtration through a PTFE filter (0.45 μm). The filtrate was taken for chromatographic analysis. The separation of sugars was performed on a ZORBAX Carbohydrate (5 μm, 4.6 × 150 mm, Agilent, Santa Clara, CA, USA) and a ZORBAX Reliance Cartridge guard column connected to an Agilent 1220 Infinity HPLC system with a 1260 refractive index detector (RID). The elution was performed at a flow rate of 1.0 mL/min at 25 °C with a mobile phase composed of acetonitrile and water (80:20 *v*/*v*). The concentration of sugars in the sample was deduced using a calibration curve constructed by plotting the peak area against five different concentrations for each sugar. The peak corresponding to different sugars in the sample was confirmed by a comparison of retention time with that of the standards.

#### 4.3.10. HPLC Determination of Organic Acids

HPLC determination of organic acids was performed on an HPLC system (Agilent 1220, Agilent Technology, Santa Clara, CA, USA), with a binary pump and UV-Vis detector (Agilent Technology, USA). Separation was performed on an Agilent TC-C18 column (5 μm, 4.6 mm × 250 mm) at 25 °C. Twenty microliters of the extract, prepared as described in 4.3.9., were injected and eluted (1.0 mL/min) isocratically with a 25 mM solution of K_2_HPO_4_ in water, whose pH was finely adjusted to 2.4 with H_3_PO_4_. The UV detector was set at 210 nm. The concentration of each organic acid in the sample was calculated using a calibration curve obtained by using five different concentrations for each acid. The peak corresponding to different acids was confirmed by a comparison of the retention time with that of the standards.

#### 4.3.11. HPLC Determination of Phenolic Components

Before analysis, the finely ground (<500 µm) sample (0.5 g) was mixed with 40 mL of a solvent containing 60% ethanol in 0.5% formic acid, and further constantly stirred at room temperature for 1 h. The mixture was centrifuged (6000× *g*, 20 min) and the supernatant was then used for HPLC analysis, total polyphenols, and in vitro antioxidant activity determination. Different phenolic components were determined using an HPLC system (Agilent 1220, Agilent Technology, USA), with a binary pump and UV-Vis detector (Agilent Technology, USA). Separation was performed on an Agilent TC-C18 column (5 μm, 4.6 mm × 250 mm) at 25 °C and a wavelength of 280 nm was used. The following mobile phases were used: 0.5% acetic acid (A) and 100% acetonitrile (B) at a flow rate of 0.8 mL/min. The gradient elution started with 14% B, between 6 min and 30 min, linearly increased to 25% B, and then to 50% B at 40 min. The identification of the compounds was confirmed by a comparison of retention times utilizing the standard solutions and standard calibration curves of different phenolics.

#### 4.3.12. Total Polyphenolic Content and In Vitro Antioxidant Activity

The total phenolic content was determined according to the method of Singleton and Rossi with Folin–Ciocalteu’s reagent [47]. Gallic acid (10–200 μg/mL) was employed as a calibration standard. The oxygen radical absorbance capacity (ORAC) and hydroxyl radical averting capacity (HORAC) were measured according to the methodology used by Denev et al. (2010) [48]. Both analyses were carried out on a FLUOstar OPTIMA plate reader (BMG Labtech, Ortenberg, Germany).

#### 4.3.13. Scanning Electron Microscopy

The surface and internal topography and composition of the dried non- and 25 kGy-irradiated RH fruit flesh (non-ground) were examined by a digitalized SEM Philips 515 (accelerating voltage 8 kV, magnification 500×, 0.1 mm) equipped with an Everhart–Thornley secondary and Robinson backscattered electron detector (Philips, Amsterdam, the Netherlands). An SC7620 Mini Sputter Coater (Quorum Technologies, Laughton, East Sussex, UK) was used for coating specimens prior to examination with the microscope. The preparation and examination of the specimens were carried out in the Laboratory of Electron Beam Microscopy, Institute of Optical Materials and Technologies “Acad. Jordan Malinowski” (Bulgarian Academy of Sciences, Sofia, Bulgaria).

### 4.4. Extraction of the Polysaccharide Fraction

The ground sample (dried non-, 10 kGy-, and 25 kGy-irradiated fruit flesh of RH) was extracted with distilled water at 80 °C for 1 h, mixing the plant material and solvent in a ratio of 1 to 25 (*w*/*v*). After cooling, the solid was separated from the liquid through centrifugation (4428× *g* for 30 min at 20 °C) and an additional Büchner funnel filtration (filter paper, KA-4, OP Papírna, s.r.o., Olšany, Czech Republic). To the filtrate, 3.0 volumes of 96 % (*v*/*v*) cold ethanol were added, and after standing overnight at 4 °C, the resulting precipitate was collected by centrifugation (20 min, 4 °C, 3490× *g*). Then, the precipitate was re-dissolved in distilled water, dialyzed (mwco 12,000–14,000 Da; VISKING^®^, SERVA Electrophoresis) for 72 h against distilled water (4 °C), and freeze-dried.

### 4.5. Endo-Polygalacturonase (endo-PG) Hydrolysis of the Polysaccharide Fraction

Initially, PSs (2 mg/mL), isolated as described in 4.4., were dissolved in 50 mM of sodium acetate buffer (pH 5.2) and incubated for 24 h at 40 °C with *endo*-PG-I-M2 (EC 3.2.1.15; *Aspergillus aculeatus*; 5000 U/mL; 0.16 U/mL [S]) purchased from Megazyme International Ltd. (Bray, Co., Wicklow, Ireland). Finally, the enzyme was inactivated at 95 °C for 5 min, and after cooling the digest was centrifuged at 18.187× *g*, at 5 °C for 10 min. The supernatant obtained was further analyzed for the molecular weight distribution.

### 4.6. Physico-Chemical Characterization of Polysaccharides

#### 4.6.1. General Analytical Methods

For the estimation of the total uronic acid content of the PSs, appropriately diluted solutions of PSs were directly assayed by the colorimetric method as described above (4.3.3.). For the estimation of the degree of methyl esterification, PSs (1 mg/mL) were saponified (0.5 M NaOH) and after neutralization (1.0 M HCl) the quantity of methanol was evaluated using a combined alcohol oxidase/4-amino-5-hydrazino-1,2,4-triazole-3-thiol (Purpald^®^) method [49]. The analysis was carried out according to the methodology used by Anthon and Barrett [50]. The acetyl content was determined photometrically by the hydroxamic acid reaction method of McComb and McCready, using *β*-d-glucose pentaacetate (24–120 μg/mL) as a standard [51]. The degrees of methylation and acetylation were expressed as moles of methyl esters or acetyl groups per 100 moles of uronic acid, respectively. The protein content was estimated by the dye-binding method of Bradford using Coomassie^®^ Brilliant blue G-250 dye (Amresco^®^) and bovine serum albumin as a standard [52].

#### 4.6.2. Monosaccharide Composition Analysis

The monosaccharide composition analysis of non-irradiated and 25 kGy-irradiated PSs was carried out according to the method of Honda et al. as modified by Yang et al. [53,54]. In brief, the PSs were hydrolyzed (4M TFA, 8 h at 110 °C) and then the released monosaccharides were derivatized employing 1-phenyl-3-methyl-5-pyrazolone (PMP) to UV-absorbing products. The resulting PMP derivatives were separated on an Agilent 1220 HPLC system with a UV detector (250 nm). The separation was conducted on an Agilent TC-C18 (4.6 × 250 mm, 5 μm) column with a mobile phase consisting of 50 mM phosphate buffer (Na_2_HPO_4_-NaH_2_PO_4_, pH 6.9) and acetonitrile using a gradient elution [54].

#### 4.6.3. Molecular Weight Distribution Analysis

PSs samples (initial and *endo*-PG-digested) were analyzed using an HPSEC Agilent 1220 Infinity LC system coupled with a 1260 RID detector using an Agilent Bio SEC-3 (300 Å, 4.6 × 300 mm, 3 μm) column. The elution was performed with a mobile phase of 150 mM NaH_2_PO_4_ (pH 7.0) employing a flow rate of 0.5 mL/min. Pullulan standards (Shodex 159 standard P-82 kit, Showa Denko, Kawasaki, Japan) with molecular weights in the range of 0.59 × 10^4^ to 78.8 × 10^4^ g/mol were used for the construction of a standard curve by plotting the logarithm of the molecular weight and retention time.

### 4.7. FT-IR Spectroscopy

The sample (4 mg) was mixed with spectroscopic grade KBr and was then pressed into a pellet. FT-IR spectrum was collected on a Nicolet Avatar 330 (Thermo Electron Corp., Waltham, MA, USA) spectrometer. The spectrum was recorded over a wavenumber range of 4000–400 cm^−1^ at 132 scans with a spectral resolution of 4 cm^−1^. The analysis and processing of the obtained spectrum were performed using the Spekwin32 software (version 1.71.5).

### 4.8. Statistical Analysis

Measurements were carried out in triplicate. The results are presented as a mean value of parallel determinations ± standard deviation and were compared by the Student’s *t*-test (MS Excel 2010 software).

## 5. Conclusions

For the first time, an in-depth study entirely devoted to the examination of the effect of gamma irradiation on phytochemical constituents of dried RH fruits was carried out. What is more, the current study is the first provide valuable insight into the cell wall PS constituents of irradiated RH fruits. To our knowledge, the gathered data for carbohydrate, organic acids, and lipid composition are also reported for the first time in this study. The results of our experiment indicate that the gamma irradiation of dried RH does not significantly change the content of fructose, most organic acids, fatty acids, *β*-carotene, and total polyphenols. By contrast, cellulose and pectin, which constituted the majority of plant cell walls, were adversely affected proportionately to the irradiation dose (0 < 10 < 25 kGy), which was indicated by changes in the yield, sugar content, and molecular weight distribution pattern. Therefore, our findings could be interpreted in various ways. On the one hand, it seems reasonable to suggest that the exposition of dried RH fruits on doses less than or even equal to 25 kGy have a negligible effect on the lower molecular weight constituents (such as some lipids, sugars, pigments, organic acids, and polyphenols). This means that some nutritional and functional properties of food predetermined by these components would not significantly change. On the other hand, it seems that PSs constituents may be affected by doses even lower than 10 kGy. However, the fruits may change their textural properties when their PS components are changed/decomposed, thus exerting an influence on the way the fruit is further technologically processed. In addition, there is an expectation that pectin would change its jellifying properties. Nevertheless, it could be compromised that the exposition of dried RH up to 10 kGy is safe and can be used to decontaminate fruits, which are very often used as an ingredient in fruit-based teas and functional beverages.

## Figures and Tables

**Table 1:** Organic acid composition of non-irradiated, 10 kGy- and 25 kGy-irradiated RH fruits (mg/100 g dw).

Organic Acid	Non-Irradiated	10 kGy	25 kGy
Quinic acid	1344.7 ± 187.5 ^ab^ *	1568.0 ± 48.4 ^a^	1376.8 ± 81.0 ^b^
Malic acid	111.8 ± 6.4 ^b^	133.0 ± 3.1 ^a^	118.7 ± 9.2 ^b^
Ascorbic acid	623.5 ± 13.6 ^a^	646.0 ± 12.2 ^a^	654.1 ± 19.0 ^a^
Citric acid	2768.7 ± 196.6 ^b^	3299.9 ± 55.8 ^a^	3536.7 ± 303.5 ^a^
*α*-Ketoglutaric acid	<50 ^b^	75.8 ± 5.1 ^a^	71.5 ± 5.2 ^a^
Succinic acid	70.8 ± 1.6 ^b^	79.9 ± 10.4 ^b^	107.6 ± 7.3 ^a^
Tartaric acid	49.2 ± 4.9 ^a^	50.7 ± 1.2 ^a^	34.2 ± 1.8 ^b^

* Different letters within each row indicate statistically different values (*p* < 0.05).

**Table 2:** Yield and lipid composition of seed oil extracted from non-irradiated, 10 kGy-, and 25 kGy-irradiated RH fruits.

	Non-Irradiated	10 kGy	25 kGy
Yield, *w/w*%	6.3 ± 0.1 ^a^ *	6.5 ± 0.6 ^a^	6.4 ± 0.8 ^a^
Saturated fatty acids (rel. %)			
Palmitic acid (16:0)	3.7 ± 0.1 ^a^	3.6 ± 0.1 ^a^	3.7 ± 0.1 ^a^
Stearic acid (18:0)	2.2 ± 0.0 ^a^	2.2 ± 0.0 ^a^	2.2 ± 0.0 ^a^
Arachidic acid (20:0)	0.9 ± 0.1 ^a^	0.9 ± 0.1 ^a^	0.9 ± 0.1 ^a^
Behenic acid (22:0)	0.1 ± 0.0 ^a^	0.1 ± 0.0 ^a^	0.1 ± 0.0 ^a^
Unsaturated fatty acids (rel. %)			
Palmitoleic acid (16:1)	0.2 ± 0.0 ^a^	0.2 ± 0.0 ^a^	0.2 ± 0.0 ^a^
Oleic acid (18:1)	16.1 ± 0.5 ^a^	16.5 ± 0.6 ^a^	16.4 ± 0.5 ^a^
*cis*-Vaccenic acid (18:1)	0.5 ± 0.0 ^a^	0.5 ± 0.0 ^a^	0.5 ± 0.0 ^a^
Linoleic acid (18:2)	54.8 ± 1.2 ^a^	54.4 ± 1.3 ^a^	54.6 ± 1.5 ^a^
*α*-Linolenic acid (18:3)	21.1 ± 0.8 ^a^	21.3 ± 0.9 ^a^	21.0 ± 1.0 ^a^
Gondoic acid (20:1)	0.4 ± 0.0 ^a^	0.4 ± 0.0 ^a^	0.4 ± 0.0 ^a^
Total carotenoids (mg/kg)	365 ± 12 (816 ± 30) ^a^ **	336 ± 35 (818 ± 26) ^a^	350 ± 10 (825 ± 30) ^a^
*β*-Carotene (mg/kg)	105 ± 7 ^a^	110 ± 13 ^a^	115 ± 11 ^a^
*α*-Tocopherol (mg/kg)	364 ± 28 ^a^	295 ± 5 ^b^	272 ± 1 ^c^
*γ*-Tocopherol (mg/kg)	1042 ± 16 ^a^	937 ± 17 ^ab^	914 ± 9 ^b^
Acid value (mg KOH/g)	1.3 ± 0.0 ^b^	1.3 ± 0.0 ^b^	1.7 ± 0.0 ^a^
conj. Dienes (A_232_, 1%)	2.4 ± 0.1 ^c^	3.0 ± 0.1 ^b^	4.3 ± 0.8 ^a^
conj. Trienes (A_268_, 1%)	0.6 ± 0.0 ^b^	0.6 ± 0.0 ^b^	0.8 ± 0.0 ^a^

* in each row, no statistically significant changes are observed for the yield and each fatty acid. For the other components, different letters within each row denote statistically significant differences between the values (*p* < 0.05); ** Values in brackets represent the total carotenoid content of RH fruit flesh.

**Table 3:** Phenolic constituents and in vitro antioxidant activity of non-irradiated, 10 kGy-, and 25 kGy-irradiated RH fruits.

		Non-Irradiated	10 kGy	25 kGy
Total phenolics, mg GAE/100 g dw		13,148 ± 775 ^a^ *	13,840 ± 625 ^a^	13,677 ± 646 ^a^
Phenolic constituents, mg/100 g dw	Chlorogenic acid	139.7 ± 2.9 ^a^	129.9 ± 2.7 ^a^	128.8 ± 1.9 ^b^
	Quercetin	13.9 ± 0.6 ^b^	18.9 ± 0.8 ^a^	18.3 ± 0.3 ^a^
	Quercetin 3-*O*-*β*-D-glucopyranoside	68.2 ± 0.6 ^a^	67.0 ± 2.6 ^ab^	63.9 ± 0.5 ^b^
	Rutin	355.7 ± 2.8 ^a^	350.7 ± 4.9 ^ab^	347.1 ± 2.7 ^b^
	Catechin	645.4 ± 21.7 ^a^	633.3 ± 23.1 ^a^	661.6 ± 9.2 ^a^
	Epicatechin	425.2 ± 5.8 ^a^	426.1 ± 15.1 ^a^	429.1 ± 1.5 ^a^
Antioxidant activity	ORAC (µmol TE/g)	3890 ± 282 ^a^	3862 ± 198 ^a^	3899 ± 208 ^a^
	HORAC (µmol GAE/g)	593 ± 68 ^a^	573 ± 55 ^a^	599 ± 50 ^a^

* Different letters within each row denote the statistically significant differences between values (*p* < 0.05); ORAC—oxygen radical absorbance capacity; TE—Trolox equivalents; HORAC—hydroxyl radical averting capacity; and GAE—gallic acid equivalents.

**Table 4:** Yield and chemical characteristics of alcohol-insoluble solids, initial RH fruits, and polysaccharides (*w*/*w*%).

	Characteristic Parameter	Non-Irradiated	10 kGy	25 kGy
Alcohol-insoluble solids	Yield	44.1 ± 0.2 ^a^ *	42.5 ± 0.3 ^b^	40.6 ± 0.2 ^c^
Initial RH fruits	Uronic acids	10.5 ± 0.5 ^a^	9.5 ± 0.5 ^a^	10.1 ± 0.2 ^a^
	% of total uronic acids extracted	51.0	63.4	70.5
	Cellulose	7.0 ± 0.1 ^a^	6.0 ± 0.2 ^b^	5.7 ± 0.0 ^c^
	Crude protein (N×6.25)	1.7	-	-
Polysaccharides	Yield	10.8 ± 0.2 ^c^	12.8 ± 0.1 ^b^	13.4 ± 0.3 ^a^
	Uronic acids	49.5 ± 1.5 ^b^	47.5 ± 1.0 ^b^	53.1 ± 0.6 ^a^
	Degree of methyl-esterification **	69.6 ± 0.5 ^a^	70.5 ± 1.0 ^a^	70.1 ± 0.7 ^a^
	Degree of acetylation ** (Acetyl content)	10.5 ± 0.5 ^b^ (1.3)	13.0 ± 0.2 ^a^ (1.5)	13.3 ± 0.3 ^a^ (1.7)
	Protein	1.7 ± 0.2 ^a^	1.6 ± 0.0 ^a^	1.7 ± 0.1 ^a^

* Different letters in each row denote the statistically significant difference between values (*p* < 0.05); ** moles methanol or acetyl per 100 moles of uronic acids (mol%).

**Table 5:** Monosaccharide composition of the polysaccharides isolated from non-irradiated and 25 kGy-irradiated RH fruits (mol%).

Monosaccharide	Non-Irradiated	25 kGy
Neutral sugars		
Rhamnose	7.1 ± 1.0 ^a^ *	8.8 ± 0.8 ^a^
Arabinose	9.6 ± 1.0 ^a^	11.0 ± 1.2 ^a^
Galactose	6.5 ± 0.7 ^a^	5.7 ± 1.0 ^a^
Glucose	11.2 ± 1.3 ^a^	7.5 ± 0.4 ^b^
Mannose	0.1 ± 0.0 ^a^	0.0 ± 0.0 ^a^
Fucose	1.5 ± 0.6 ^b^	4.2 ± 1.0 ^a^
Uronic acids		
Galacturonic acid	56.5 ± 1.5 ^a^	58.8 ± 0.5 ^a^
Glucuronic acid	7.4 ± 0.4 ^a^	3.9 ± 1.2 ^b^

* Different letters in each row denote the statistically significant difference between values (*p* < 0.05).

## Data Availability

Not applicable.

## Sample Availability

Samples of the compounds are available from the authors.
